# Crystal structure of a DNA aptamer bound to *Pv*LDH elucidates novel single-stranded DNA structural elements for folding and recognition

**DOI:** 10.1038/srep34998

**Published:** 2016-10-11

**Authors:** Sung-Jin Choi, Changill Ban

**Affiliations:** 1 Department of Chemistry, Pohang University of Science and Technology (POSTECH), Pohang, 790-784, South Korea

## Abstract

Structural elements are key elements for understanding single-stranded nucleic acid folding. Although various RNA structural elements have been documented, structural elements of single-stranded DNA (ssDNA) have rarely been reported. Herein, we determined a crystal structure of *Pv*LDH in complex with a DNA aptamer called pL1. This aptamer folds into a hairpin-bulge contact by adopting three novel structural elements, viz, DNA T-loop-like motif, base–phosphate zipper, and DNA G·G metal ion zipper. Moreover, the pL1:*Pv*LDH complex shows unique properties compared with other protein:nucleic acid complexes. Generally, extensive intermolecular hydrogen bonds occur between unpaired nucleotides and proteins for specific recognitions. Although most protein-interacting nucleotides of pL1 are unpaired nucleotides, pL1 recognizes *Pv*LDH by predominant shape complementarity with many bridging water molecules owing to the combination of three novel structural elements making protein-binding unpaired nucleotides stable. Moreover, the additional set of *Plasmodium* LDH residues which were shown to form extensive hydrogen bonds with unpaired nucleotides of 2008s does not participate in the recognition of pL1. Superimposition of the pL1:*Pv*LDH complex with hLDH reveals steric clashes between pL1 and hLDH in contrast with no steric clashes between 2008s and hLDH. Therefore, specific protein recognition mode of pL1 is totally different from that of 2008s.

Folded, single-stranded nucleic acid (ssNA) molecules play vital roles in biological processes such as the maintenance of linear DNA length^1^, RNA processing, gene expression^2^ and ssNA virus genomes^3^. Artificially synthesised ssNA molecules, called aptamers, have emerged as good alternatives to antibodies for recognising specific molecules for the diagnosis and treatment of diseases^4^,^5^. To perform their functions, ssNA molecules (ssNAs) fold into appropriate structures. Tertiary structures are three-dimensional arrangements of secondary structures. ssNA secondary structures are classified into base-paired regions (helices) and non-paired regions (loops). Interestingly, more stable structures, which are motifs constrained via tertiary interactions, are ubiquitously observed in RNA^6^,^7^. These structures are defined as structural elements here and are classified into secondary structural elements, tertiary interaction elements and metal-binding elements. RNA hairpin loop motifs and internal loop motifs are classified as secondary structural elements. Tertiary interaction elements include loop:loop, loop:helix and helix:helix interactions such as A-minor motifs, kissing hairpin loops, pseudoknots, coaxial helices, ribose zippers, triplexes, quadruplexes, tetraloop:tetraloop receptor complexes and T-loop:D-loop complexes^8^. Metal-binding motifs serve to reduce the electrostatic repulsion of the phosphate backbone via intramolecular interactions^9^. In particular, large non-coding RNA forms highly complicated structures by adopting various structural elements^10^,^11^,^12^,^13^,^14^. Therefore, structural elements are important for understanding RNA folding. In addition, the diversity of RNA folds and 3D-structures serves as the basis for the functional diversity of RNA molecules^15^. While various RNA structural elements have been documented, ssDNA structural elements have rarely been reported because of their scarcity. Only hairpin (or distorted hairpin), G-quadruplex, triplex and three-stem structures of ssDNA have been determined^16^,^17^,^18^,^19^. These folded ssDNA molecules have increasingly emerged as potentially important players. In addition to the maintenance of linear DNA length by DNA telomeres that fold into G-quadruplexes, some transcriptional gene expression is regulated by DNA tertiary structures such as *i*-motifs, G-quadruplexes and hairpins near promoter regions^20^. Apart from artificially synthesised DNA aptamers, DNA deoxyribozymes have been artificially identified for a broad spectrum of applications^21^,^22^,^23^,^24^. Thus, the structural characterisation of a large range of ssDNA structures is required to identify the specific structural elements that govern their specific functions and specific target recognition.

In living cells, ssNAs function by interacting with various proteins. Protein nucleic acid-binding regions are primarily positively charged via electrostatic complementarity to compensate for the negatively charged phosphate backbone. In addition to charge complementarity, unpaired nucleotides play an important role in protein recognition. In particular, unpaired nucleotides in loops and bulges are involved in specific protein recognition through extensive hydrogen bonds (H-bonds) between bases and proteins^25^,^26^,^27^. For example, in ssNAs folded into G-quadruplexes and hairpins, hydrogen bonding predominates between unpaired bases and protein surfaces for protein recognition. To further understand the various interactions involved between ssNAs and proteins, it is critical to determine the structures of protein:ssNA complexes. However, besides modified DNA such as SOMAmers^28^,^29^,^30^, only five non-redundant structures of protein:DNA aptamer complexes^31^,^32^,^33^,^34^,^35^,^36^ have been identified.

Previously, we identified DNA aptamers for the specific recognition of *Plasmodium* lactate dehydrogenase (pLDH) for the diagnosis of malaria disease in human blood samples via impedance measurements and colorimetry. The dissociation constants of the DNA aptamer pL1 for the recognition of *P. vivax* lactate dehydrogenase (*Pv*LDH) and *P. falciparum* LDH (*Pf*LDH) were found to be 16.8 nM and 38.7 nM, respectively^37^,^38^. The crystal structure of *Pf*LDH in a complex with the DNA aptamer 2008s was determined by another group. The 2008s:*Pf*LDH complex shows 1:2 stoichiometry in which 2008s folds into a distorted hairpin structure. The DNA aptamer 2008s discriminates between pLDH and human lactate dehydrogenase (hLDH) via extensive H-bonds with the additional set of pLDH residues, referred to as ASPR, which is not present in hLDH^35^. Although the dissociation constants of 2008s for the recognition of pLDH are similar to those of pL1 for the recognition of pLDH, the sequence and secondary structure of 2008s are different from those of pL1. Therefore, we sought to further understand the unique 3D-structures and interface properties of pL1 for the specific recognition of pLDH that are different from those of the 2008s:*Pf*LDH complex. Some folded ssNA structures have elucidated novel structural elements that have served to increase the understanding of various ssNA folds. In this study, we determined high-resolution structures of the pL1:*Pv*LDH complex and apo-*Pv*LDH by X-ray crystallography in order to understand novel ssDNA structural elements, ssDNA folding and interface features for specific pLDH recognition. pL1 folds into a hairpin-bulge contact by adopting three novel structural elements defined as a T-loop-like motif, base–phosphate (BPh) zipper and DNA G·G metal ion zipper. Interestingly, consecutive BPh interactions, referred to as BPh zippers, have not been observed in RNA or double-stranded DNA structures. In addition to novel ssDNA structures, the pL1:*Pv*LDH interface demonstrated interesting properties. Unlike the general interactions of unpaired nucleotides, the combination of three novel structural elements appears to enable protein-binding unpaired nucleotides to be stable and unfavourable to extensive intermolecular H-bonds. Therefore, pL1 recognises *Pv*LDH predominantly via shape complementarity between inflexible regions and many bridging water molecules (bridging waters). Moreover, tight packing of the pL1:*Pv*LDH interface aids in the discrimination of pLDH from hLDH, which is incompatible with shape complementarity. Thus, the specific recognition mode of pL1 appears to be completely different from that of 2008s.

## Results and Discussion

### Dimeric *Pv*LDH binds to one DNA aptamer, folding into a unique hairpin-bulge contact

**Figure 1:**
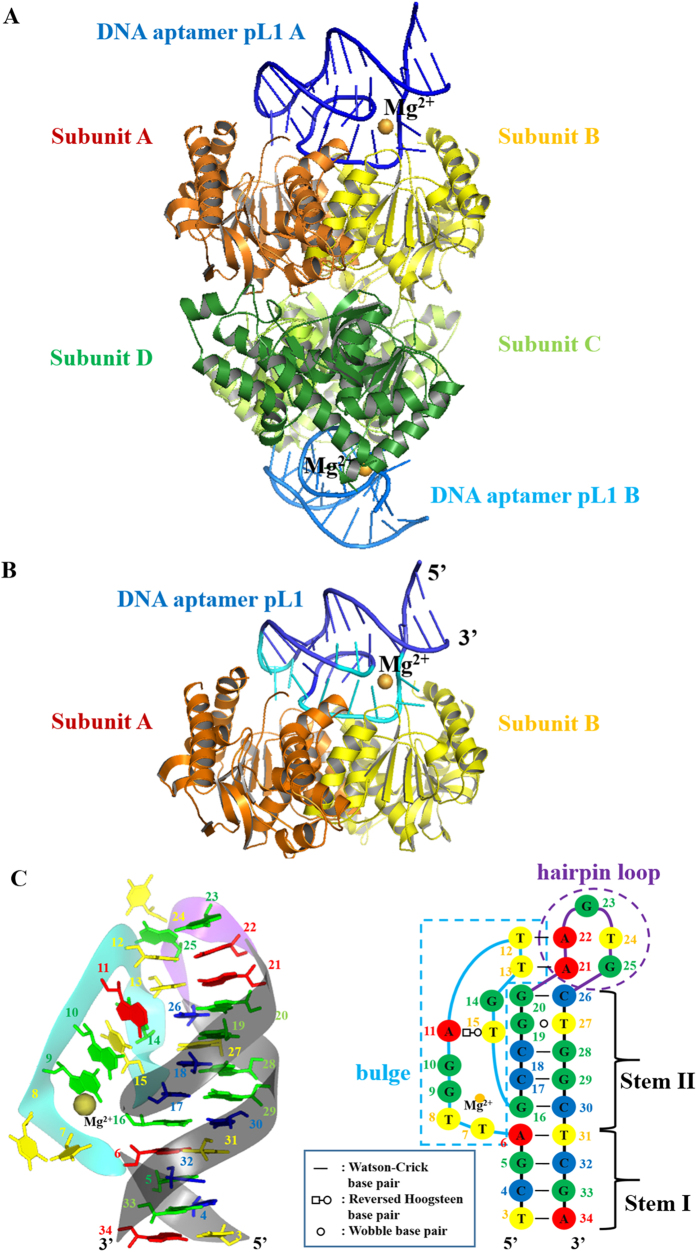
Overall structures of *Pv*LDH in complex with pL1 that folds into a hairpin-bulge contact. Gold sphere indicates a magnesium ion. (**A**) Tetrameric *Pv*LDH interacts with two DNA aptamers. *Pv*LDH subunits A, B, C, and D are shown in orange, yellow, limon, and green, respectively. pL1 A and B are colored in blue and marine, respectively. (**B**) Dimeric *Pv*LDH interacts with one DNA aptamer. *Pv*LDH subunits A and B are shown in orange and yellow, respectively. *Pv*LDH-contacting nucleotides and non-contacting nucleotides of pL1 are shown in cyan and blue, respectively. (**C**) Structure and schematic of a DNA aptamer called pL1. Ribbon representation of pL1 and the secondary structure derived from the tertiary structure. Strands forming the bulge, hairpin loop, and stems are shown in cyan, purple, and black, respectively. Adenines, guanines, thymines, and cytosines are shown in red, green, yellow, and blue respectively. Nucleotides at the bulge and hairpin loop are boxed with cyan and purple dashed lines, respectively.

The pL1:*Pv*LDH complex was crystallised under two different conditions. The crystals belonged to *P*2_1_2_1_2 and *P*4_1_2_1_2 at 1.9 Å and 1.71 Å resolution, respectively (Table S1). Two aptamers were bound to tetrameric *Pv*LDH in an asymmetric unit of *P*2_1_2_1_2 (Fig. 1A). One aptamer was bound to dimeric *Pv*LDH in an asymmetric unit of *P*4_1_2_1_2 (Fig. 1B). Despite different space groups, the crystal packing and interface of the pL1:*Pv*LDH complexes were similar (Fig. 1A,B). Therefore, all descriptions provided below are based on the pL1:*Pv*LDH complex structure belonging to *P*4_1_2_1_2.

Electron density of pL1 was clearly observed (Fig. S1). pL1 folds into a unique hairpin-bulge contact in complex with *Pv*LDH. The main elements of pL1 include two stems, an intervening bulge and a hairpin loop (Fig. 1C). The two stem elements are stabilised by stacking base pairs. Four Watson–Crick-type base pairs (T3-A34, C4-G33, G5-C32 and A6-T31) are stacked as a B-form helical DNA duplex at Stem I. Stem II comprises four Watson–Crick-type base pairs (G16-C30, C17-G29, C18-G28 and G20-C26) and one wobble base pair (G19·T27). In contrast to intervening bulges or internal loops that result in distorted helices, coaxial stacking occurs at the interface between the terminal base pairs of Stem I and Stem II. Owing to coaxial stacking, unpaired nucleotides of the bulge are pushed out of the stems. However, the hairpin and intervening bulge are stabilised by the hairpin-bulge contact. Two Watson–Crick-type base pairs are created (A22-T12 and A21-T13) between the hairpin loop and bulge. Similar to RNA pseudoknots, coaxial stacking is formed at the interface between Stem II and the hairpin-bulge contact. Thus, a pseudo-continuous helix is built from Stem I to the hairpin-bulge contact by coaxial stacking (Fig. S2).

### T-loop-like motif is a novel ssDNA secondary structural element contributing as a nucleation site

The T-loop motif, a RNA hairpin motif, consists of a UA *trans*-Watson–Crick/Hoogsteen closing base pair and a U-turn sub-motif following three specific nucleotides, 5′-(G/U)NR-3′ (N: any nucleotide; R: purine). The U-turn sub-motif is stabilised by H-bonds between the first (G/U) and third (R) residues. The imino proton of the G/U interacts with the phosphate of the R residue via a BPh interaction belonging to the 5BPh interaction. Moreover, one hydrogen bond (H-bond) is formed between the 2′ hydroxyl group of R and the imino proton of the G/U residue^39^.

**Figure 2:**
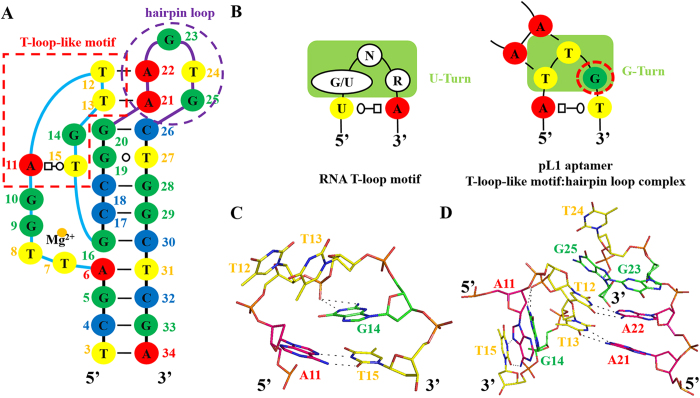
DNA T-loop-like motif induces T-loop-like motif:hairpin loop complex. (**A**) DNA T-loop-like motif and hairpin loop are in the secondary structure derived from the teritary structure. Nucleotides at the T-loop-like motif and hairpin loop are boxed with red and purple dashed lines, respectively. (**B**) Comparision between the RNA T-loop motif and DNA T-loop-like motif inducing the hairpin-bulge contact. N represents any nucleotides; R stands for purine. Nucleotides at T-turn and G-turn are boxed in yellowish green. Guanines, adenines, thymines, and cytosines are shown in green, red, yellow, and blue, respectively. (**C**) Stick diagram of the DNA T-loop-like motif. Guanines, adenines, and thymines are shown in green, pink, and yellow, respectively. (**D**) Stick diagram of the T-loop-like motif:hairpin loop complex.

To date, no study has reported a similarity between ssDNA structural elements and the RNA T-loop motif. Interestingly, the apical bulge of pL1 is stabilised by a novel structural element similar to the RNA T-loop motif. Of the apical unpaired nucleotides of the bulge, including 5′-A11, T12, T13, G14 and T15-3′, 5′-A11 interacts with T15 via a reversed Hoogsteen base pair. In other words, the TA *trans*-Watson–Crick/Hoogsteen closing base pair occurs within the bulge instead of the TU *trans*-Watson–Crick/Hoogsteen closing base pair of the RNA T-loop. T12, T13 and G14 form sharp bend-like U-turn sub-motifs. However, the second (T13) and third (G14) nucleotides are stabilised by H-bonds, unlike RNA U-turn motifs where H-bonds occur between the first and third nucleotides. At the sharp turn containing 5′-T12, T13 and G14-3′, there are no H-bonds between the sugars and bases because of the absence of 2′-hydroxyl DNA groups. However, the 1-endocylic amine group and 2-exocyclic amino group of G14 interact with the phosphate oxygen group of T13 through a 4BPh interaction. Thus, the third guanine nucleotide (G14) plays a critical role in the sharp bend formation (Fig. 2A–C). Moreover, the third guanine is conserved between pL1 and pL2, through which pLDH is specifically recognised^37^. Therefore, the DNA turn motif is defined as a G-turn motif (Fig. 2B).

In addition to the formation of thermodynamically stable structures, RNA T-loop motifs contribute as nucleation sites, inducing long-range tertiary interactions such as T-loop:D-loop and T-loop:receptor complexes. The DNA T-loop-like motif also induces a T-loop-like motif:hairpin loop complex via a hairpin-bulge contact. The T-loop:D-loop and T-loop:receptor complexes contain one Watson–Crick-type base pair with other non-canonical interactions. However, the hairpin-bulge contact occurs between the T-loop-like motif and hairpin loop via two Watson–Crick base pairs because of the absence of H-bonds between the sugars and bases, which confers more accessible T12 and T13 surfaces for interacting with complementary sequences (Fig. 2A,D).

### BPh zipper as a novel tertiary interaction element that stabilises loops adjacent to stems

**Figure 3:**
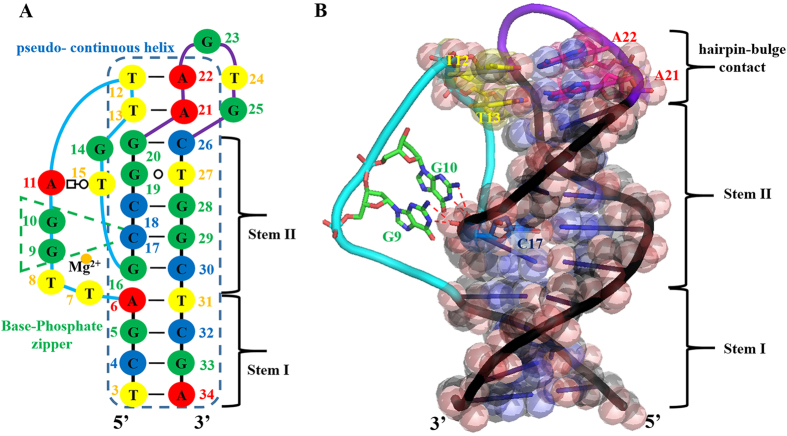
Unpaired nucleotides adjacent to helices are stabilized by the base-phosphate zipper. Strands forming the bulge, hairpin loop, and stems are shown in cyan, purple, and black, respectively. (**A**) Base-phosphate zipper and pseudo-continuous helix are in the secondary structure, which is derived from the tertiary structure. Nucleotides at the base-phosphate zipper and pseudo-continuous helix are boxed in green and blue dashed lines, respectively. Strands forming the bulge, hairpin loop, and stems are shown in cyan, purple, and black, respectively. Guanines, adenines, thymines, and cytosines are shown in green, red, yellow, and blue, respectively. (**B**) Two sequentially consecutive guanine bases interact with phosphate oxygens of the quasi-continuous helix. The pseudo-continuous helix is represented as a transparent sphere. Stick representation of the hairpin-bulge contact indicates the pseudo-continuous helix by coaxial stacking. Strands forming the bulge, hairpin, and stems are shown in cyan, purple, and black, respectively. Guanines, adenines, thymines, and cytosines are shown in green, yellow, pink, and blue, respectively.

Interactions between RNA helical and unpaired nucleotides occur via A-minor motifs, base triplets and ribose zippers. DNA unpaired nucleotides adjacent to stems are solely stabilised by base triplets owing to the formation of B-form helices and the absence of 2′-hydroxyl groups. To date, it has not been reported that nucleic acids adopt consecutive BPh interactions. Unlike previously reported motifs, consecutive BPh interactions mediate stabilisation between helical and unpaired nucleotides from the region between the bulge to Stem II of pL1 (Fig. 3). Of the nine unpaired nucleotides (T7, T8, G9, G10, A11, T12, T13, G14 and T15) of the bulge, five unpaired nucleotides (A11, T12, T13, G14 and T15) are stabilised by the T-loop-like motif (Fig. 2A). Of the remaining four unpaired nucleotides (T7, T8, G9 and G10), two nucleotides (T7 and T8) are looped-out. However, G9 and G10 are adjacent to Stem II, with unpaired G10 being stacked above unpaired G9 but below T15 within the bulge (Fig. 1C). Interestingly, the two unpaired nucleotides G9 and G10 interact with the 1-phosphate and 2-phosphate oxygens of C17, respectively. Both the 1-endocylic amine group and 2-exocyclic amino group of G9 and G10, respectively, contribute as H-bond donors to the phosphate oxygens via 4BPh interactions (Fig. 3B). Therefore, sequentially consecutive BPh interactions between stems and unpaired nucleotides form the BPh zipper. Interestingly, the BPh zipper is a unique ssDNA structural element that differs from RNA structural elements because RNA does not use consecutive BPh interactions. Instead, RNA molecules favour the use of 2′-hydroxyl groups. In addition to DNA T-loop-like motifs, 4BPh interactions are observed in BPh zippers. The 4BPh interaction is powerful because the 1-phosphate oxygen forms two H-bonds with the 1-endocylic amine group and 2-exocyclic amino group of guanine^40^. Therefore, exclusive ssDNA structural elements appear to favour 4BPh interactions to compensate for the lack of 2′-hydroxyl groups.

### Stabilisation of the bulge and BPh zipper by a novel DNA G·G metal ion zipper

**Figure 4:**
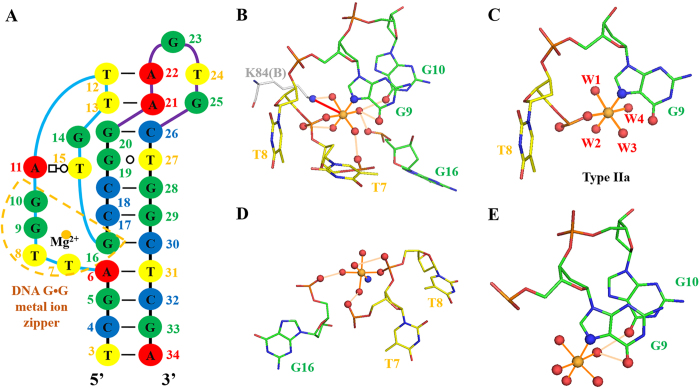
DNA G·G metal ion zipper of ssDNA. Magnesium ions are shown in gold. Inner-sphere interactions and outer-sphere H-bonds are shown in orange and wheat lines, respectively. Interacting atoms are shown as ball and stick. (**A**) DNA G·G metal ion zipper of ssDNA is boxed in gold dashed lines. (**B**) All nucleotides forming the novel magnesium ion-binding site are represented by sticks. Out-sphere ligand of Lys84B is represented by a grey stick. Red arrow indicates a salt bridge. (**C**) Nucleotides and waters forming the inner sphere represent Type IIa coordination. (**D**) Nucleotides forming the same coordination pattern as the RNA metal zipper. (**E**) Nucleotides forming the same coordination pattern as the RNA G·G metal binding site.

The metal-binding site was observed in pL1; validation of this pL1 magnesium-binding site was performed with the CheckMyMetal web server (http://csgid.org/csgid/metal_sites). All parameters were acceptable for magnesium ions (Fig. S3, Table S2). The metal-binding site of pL1 was found to differ from G-quadruplexes, well-known metal-binding ssDNA structures. The phosphate backbone of the bulge region (T7, T8, G9 and G10) is sharply bent at approximately 90°. To stabilise the bent bulge, the magnesium ion holds the highly bent conformation of the backbone (Fig. 4A,B). The phosphate oxygen of T8, the 7-imino group of G9 and four waters are present in the inner sphere of the magnesium ion, in the octahedral geometry. Of the classified magnesium ion-binding sites in the large ribosomal subunit of RNA, Type IIa shows an orthogonal geometric arrangement of two inner-sphere atoms of RNA with four waters^41^. The magnesium ion-binding site of pL1 belongs to the same geometric arrangement of Type IIa (Fig. 4C). To reduce repulsion, nucleotide atoms at the bulge and Stem II are directly or indirectly coordinated with the magnesium ion. The magnesium ion is directly linked with one 1-phosphate oxygen of T8. Out-sphere atoms are three 2-phosphate oxygens of T7, T8 and G16, although the phosphate of G16 at Stem II is distant from the phosphates of T7 and T8 at the bulge. A magnesium ion is bound through the outer sphere by two distant phosphates in the RNA metal ion zipper. Interestingly, this motif was only observed in rRNA (The term ‘distant’ means that two phosphates come from different chains or are separated by at least seven nucleotides in the same chain)^9^. This outer-sphere-coordinating pattern is similar to that of pL1 (Fig. 4D). Moreover, the oxygen atoms of 6-oxo groups at G9 and G10 are outer-sphere-coordinating atoms. This is the same outer-sphere-coordinating pattern of the RNA G·G metal-binding site in which two sequentially consecutive guanine moieties are connected to the magnesium ion^9^ (Fig. 4E). Considering all coordination patterns, it can be said the magnesium coordination of pL1 in the outer sphere is similar to that in the RNA metal ion zipper and RNA G·G metal-binding motif. Therefore, this novel metal-binding element is defined as a DNA G·G metal ion zipper. Moreover, sequentially consecutive G9 and G10, contributing to the BPh zipper, are stabilised by the magnesium ion. In addition to contributing to ssDNA folding, the magnesium ion interacts with the ammonium group of Lys84(B) through one salt bridge (Fig. 4B).

### Nature of pL1-mediated *Pv*LDH recognition

**Figure 5:**
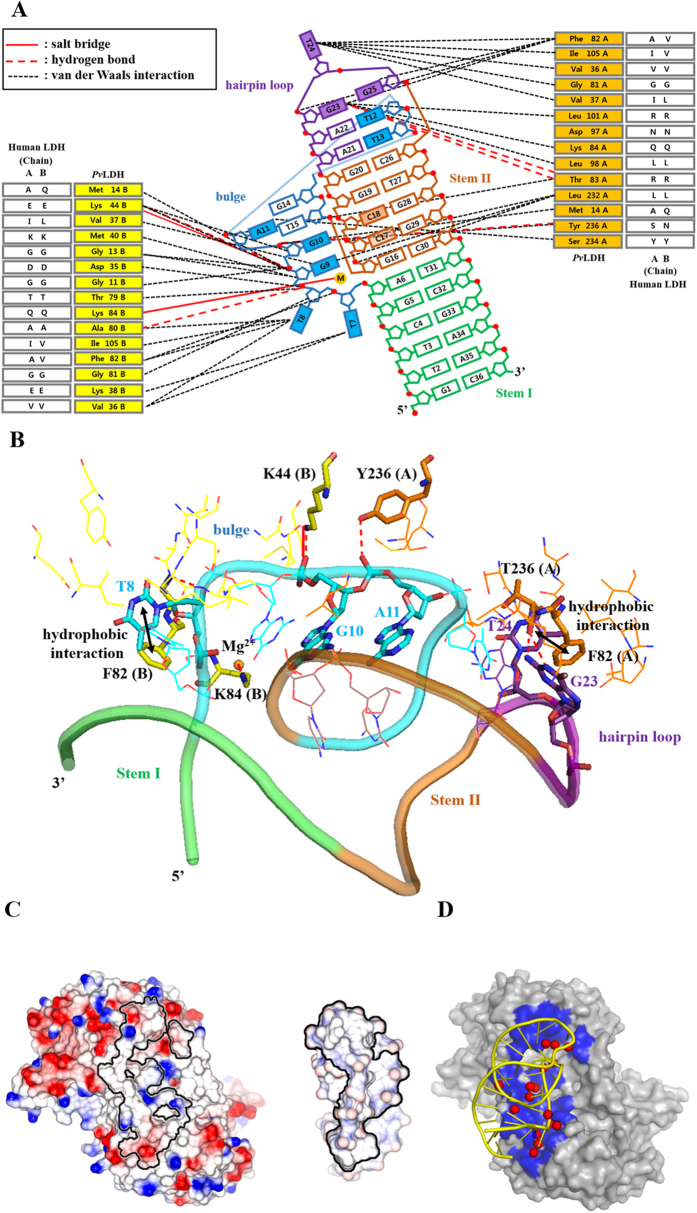
Structural analysis of the interface between pL1 and *Pv*LDH. (**A**) Schematic illustration shows the interface between the pL1 and *Pv*LDH dimer. Nucleotides in color are within 4 Å of the *Pv*LDH dimer. Solid red lines indicate salt brides; dashed red lines represent H-bonds; and dashed black lines indicate van der Waals interactions. (**B**) The box and stick diagram shows the interface between pL1 and *Pv*LDH dimer. Nucleotides participating in the H-bonds, salt bridges, and hydrophobic interactions are shown as cyan sticks. Amino acids of subunits A and B implicating hydrogen bonds, salt bridges, and hydrophobic interactions are shown as orange and yellow sticks, respectively. (**C**) Open-book view of pL1-binding interface: An electrostatic surface renders the partially basic and acidic composition of the surface. The *Pv*LDH and pL1 interface areas are outlined as lines. (**D**) Bridging water pattern of the *Pv*LDH:pL1 interface. Dimeric *Pv*LDH and pL1 are shown as surface and yellow ribbon, respectively. The interface area is in blue. Bridging waters are red spheres.

The dimeric *Pv*LDH places one pL1 in a single cluster of DNA-contacting residues that forms a concave region determined by the Cx algorithm (Fig. S4)^42^. The interface between the dimeric *Pv*LDH and pL1 buries the total accessible surface area (ASA) of 1145.25 Å^2^. The interface between pL1 and monomeric *Pv*LDH buries a solvent-accessible surface of 613.8 Å^2^ and 530.0 Å^2^ for *Pv*LDH subunits A and B, respectively. Although the same five amino acids (Met14, Val36, Val37, Gly81, Phe82 and Ile105) of both subunits participate in DNA contact, another eight amino acids at each subunit are involved in DNA interaction (Table S3). Therefore, the DNA-contacting surfaces of each subunit are asymmetric in the dimeric *Pv*LDH (Fig. 5A).

The *Pv*LDH-contacting region of pL1 is composed of two paired nucleotides at Stem II and nine unpaired nucleotides at the bulge and hairpin loop. For interactions between Stem II and pL1, Leu232(A) is solely engaged in van der Waals contacts with the sugar phosphate backbone of C17 and C18. However, the main interactions between *Pv*LDH and pL1 occur with unpaired nucleotides. Unpaired nucleotides at the bulge and hairpin loop continuously stretch away into the concave protein region because the two separated DNA regions of the bulge and hairpin loop are connected by the hairpin-bulge contact. The bulge contains *Pv*LDH-contacting nucleotides (T7, T8, G9, G10, A11 and T12), while the hairpin loop involves *Pv*LDH-contacting nucleotides (G23, T24 and G25) (Fig. 5A,B).

The *Pv*LDH-contacting unpaired nucleotides contain four unpaired and flipped nucleotides (T7, T8, T24 and G25). T8 and T24 extend into the NADH-binding clefts of subunits B and A, respectively. Unpaired and flipped nucleotides do not participate in hydrogen bonding for complex formation, with the exception of T8. Only one H-bond occurs between the 3′ oxygen of T8 and the backbone amide NH group of Gly81(B). In contrast to using H-bonds, the two bases of G23 and T8 interact with the two aromatic rings of Phe82(A, B) through hydrophobic interactions (Fig. 5B).

The unpaired G9, G10, A11, T12 and G23 nucleotides are not flipped out of their structure because they are stabilised by the three novel structural elements described above. As a result, the sugar phosphates of G9, G10, A11 and T12 are primarily exposed to the concave protein surface instead of the bases. The unpaired and not flipped nucleotides are stabilised by four H-bonds. The 1-endocyclic amine group and 2-exocyclic amino group of G23 form two H-bonds with the carbonyl backbone of Thr83(A). The 1-phosphate oxygen of A11 interacts with the phenolic hydroxyl group of Tyr236(A) by one H-bond. The 1-phosphate oxygen of G10 interacts with the ammonium group of Lys44(B). In addition to H-bonds, one salt bridge occurs between the phosphate of G10 and ammonium group of Lys44(B) to stabilise the negatively charged phosphate backbone. Therefore, a total of five intermolecular H-bonds and one salt bridge are observed at the interface between pL1 and dimeric *Pv*LDH (Fig. 5A,B, Table S3). Moreover, the magnesium ion interacts with the ammonium group of Lys84(B) through one salt bridge (Fig. 4B).

### Unique properties of the pL1:*Pv*LDH complex interface

To understand the properties of the pL1:*Pv*LDH interface, several parameters of the pL1:*Pv*LDH complex were compared with those of protein:RNA, protein:DNA and protein:DNA aptamer complexes (Table 1, S4). The buried surface area within 5 Å upon complex formation is defined as ΔASA (Å^2^). H-bonds (/100 Å^2^ ΔASA) are defined as the number of H-bonds per 100 Å^2^ ΔASA. Bridging waters (/1000 Å^2^ ΔASA) are defined as the number of water molecules forming H-bonds with both proteins and nucleic acids per 1000 Å^2^ ΔASA. All parameters were calculated by the COCOMAP web server, PISA, PyMol and CONTACT in the protein:DNA aptamer complexes. The data for the interface areas, H-bonds and bridging waters of the protein:RNA, protein:DNA and protein:protein complexes were obtained from a previous interface analysis^43^,^44^. Although most protein-binding nucleotides are unpaired nucleotides of pL1, the number of H-bonds (/100 Å^2^ ΔASA) of the pL1:*Pv*LDH complex was 0.4, which was the lowest among the mean number of the other complexes. On the other hand, extensive H-bonds are observed in ssDNA:thrombin, 2008s:*Pf*DLH and ARC1172:VWF complexes because most protein-interacting nucleotides are unpaired nucleotides. Although the main protein-binding nucleotides of anti-ATX and anti-HIV-1 RT DNA aptamers are paired nucleotides, the number of H-bonds (/100 Å^2^ ΔASA) of the ATX:RB011 and HIV-1 RT:apt-DNA complexes is higher than that of the pL1:*Pv*LDH complex. Moreover, the bridging waters (/1000 Å^2^ ΔASA) of the pL1:*Pv*LDH complex far outnumbered those of the other complexes (Table 1, S4). Fourteen bridging waters form water-mediated H-bonds between pL1 and *Pv*LDH (Fig. 5D, Table S5). To understand the contribution of bridging waters to shape complementarity, Sc values for the pL1:*Pv*LDHW and pL1:*Pv*LDH complexes were calculated. *Pv*LDHW is used to designate *Pv*LDH with bridging waters; the Sc value for pL1:*Pv*LDHW (0.720) is higher than that for pL1:*Pv*LDH (0.675) (Table S6). Therefore, fourteen bridging waters maximise shape complementarity. The electrostatic surface of the pL1-binding region is partially negatively and positively charged in *Pv*LDH, whereas that of the *Pv*LDH-binding region is mostly positively charged in pL1 (Fig. 5C). Bridging waters serve to buffer unfavourable electrostatic interactions (Fig. 5D).

### Anti-pLDH DNA aptamers and their interactions with pLDH

pL1 and 2008s are anti-pLDH DNA aptamers. In addition to the similar affinity between the DNA aptamers and pLDH, 1:2 stoichiometry is shown in both complexes. Interestingly, two flipped bases (T9 and A16 of 2008s; T8 and T24 of pL1) are stretched into the NADH-binding sites similar to the C13.18:GRK2 complex, in which A51 flips deep into the ATP-binding site^45^. Moreover, some pLDH residues interact with both pL1 and 2008s, including Gly13, Met14, Asp35, Val36, Val37, Met40, Lys44, Ala80, Gly81, Phe82, Lys84, Ile105, Leu232 and Ser234. Further, salt bridge interactions between the 1-phosphate oxygen of DNA and ammonium group of Lys44 are observed in both the complexes (Table S3).

However, unique properties in their interactions with pLDH are shown because of different DNA folding. In contrast to the metal-independent 2008s, pL1 is a metallo-DNA aptamer and the magnesium ion participates in the interaction between pL1 and *Pv*LDH. This magnesium ion, coordinated with pL1, interacts with the ammonium group of Lys84 by one salt bridge (Fig. 4B). Instead of metal ions, the ammonium group of Lys84 interacts with the phosphate of A12 in the 2008s:*Pf*LDH complex via one salt bridge. Moreover, pL1 exclusively interacts with Gly11, Lys38, Thr79, Thr83, Asp97, Leu98 and Tyr236 of *Pv*LDH, while 2008s solely interacts with Phe34, Tyr67, Ala85, Pro86, Gly87, Lys88, Ser89, Asp90, Lys91, Glu108, Val229 and Ala233 of *Pf*LDH (Table S3). In addition to different DNA-contacting residues, one cluster of DNA-contacting residues in the pL1:*Pv*LDH complex is different from the double cluster of DNA-contacting residues in which the dimeric *Pf*LDH places one 2008s (Fig. S5).

Furthermore, the main interaction mode of the pL1:*Pv*LDH complex is different from that of the 2008s:*Pf*LDH complex. Hereafter, the loop containing ASPR is referred to as FT. Although disordered in apo-*Pf*LDH, FT is well ordered in the pL1:*Pf*LDH complex because FL plays a key role in 2008s recognition via extensive H-bonds. To understand the conformational change between apo-*Pv*LDH and pL1-bound *Pv*LDH, we determined the crystal structure of apo-*Pv*LDH belonging to *I*222 at 1.6 Å resolution (Table S1). Conformational changes are subtle between the apo-*Pv*LDH dimer and pL1-bound *Pv*LDH dimer, with an overall root mean square deviation of 0.94 Å (Fig. S6). Moreover, FL is disordered in both apo-*Pv*LDH and pL1-bound *Pv*LDH. Therefore, shape complementarity between pL1 and the inflexible regions of apo-*Pv*LDH is critical for interactions between pL1 and *Pv*LDH. Here FL that is not considered in pLDHW is referred to as pLDHWwoFL. To understand the contribution of shape complementarity between aptamers and pLDHWwoFL, Sc values were calculated. Although the Sc value for pL1:*Pv*LDHW (0.720) is similar to that for 2008s:*Pf*LDHW (0.744), the Sc value for pL1:*Pv*LDHWwoFL (0.717) is higher than that of 2008s:*Pf*LDHWwoFL (0.685). Only five H-bonds and one salt bridge occur in the pL1:*Pv*LDH complex. Thus, shape complementarity and many bridging waters compensate for the lack of H-bonds in the pL1:*Pv*LDH complex. In conclusion, the main mode of pLDH recognition by pL1 is different from that by 2008s.

### Structural insight into discriminatory pLDH recognition by pL1

**Figure 6:**
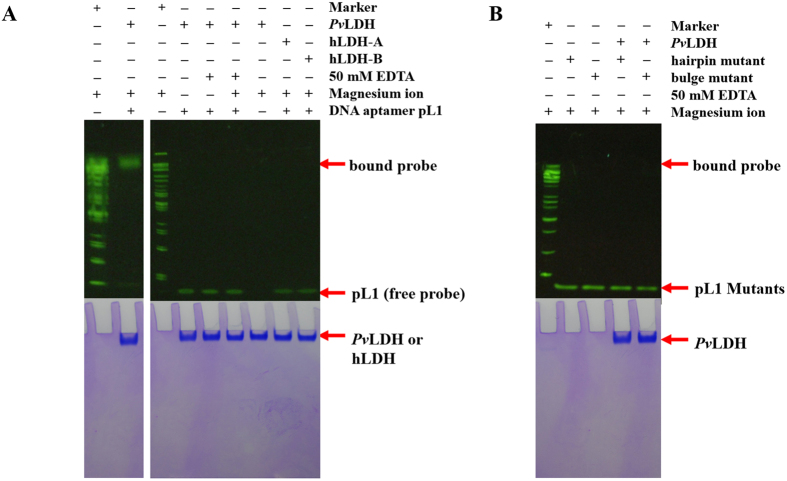
EMSA reveals the specific *Pv*LDH recognition by pL1 along with three novel structural elements. pL1 does not interact with hLDH-A and hLDH-B. Mutations of three novel structural elements result in dramatic decrease in affinities between pL1 and *Pv*LDH. (**A**) EMSA experiments to confirm the interaction between pL1 and hLDH with or without magnesium ions and EDTA. (**B**) EMSA experiments to check the interaction between pL1 mutants and *Pv*LDH.

Extensive H-bonds determine the specific recognition between 2008s and ASPR^35^. Selectivity tests using cationic surfactant-based colorimetric detection show that pL1 also discriminates pLDH from LDH-A^38^. Moreover, electrophoretic mobility shift assays (EMSAs) reveal that pL1 discriminates pLDH from hLDH-A and hLDH-B (Fig. 6A). However, ASPR does not contribute to pL1 recognition in the pL1:*Pv*LDH complex. Compared with four salt bridges and 15 H-bonds in the 2008s:*Pf*LDH complex, only one salt bridge and five H-bonds occur in the pL1:*Pv*LDH complex. Therefore, pL1 applies different strategies for specific pLDH recognition, instead of extensive H-bonds and electrostatic complementarity.

**Figure 7:**
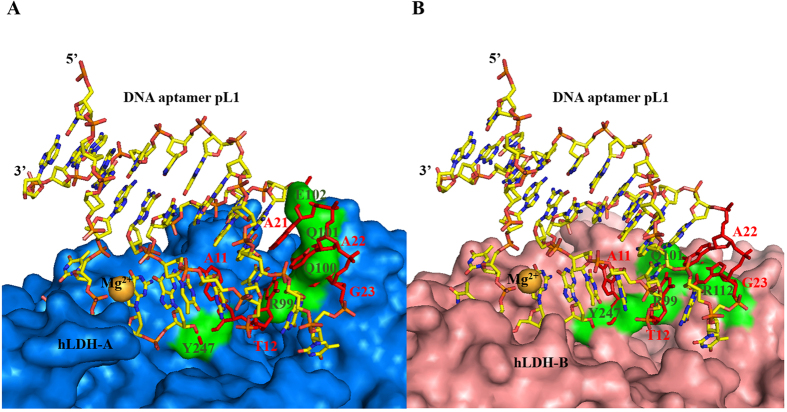
Clashes between pL1 and hLDH confers on pL1 discrimination of pLDH from hLDH. Gold spheres represent magnesium ions. Clashes occur between red pL1 nucleotides and green hLDH surfaces. (**A**) Superimposition of the pL1:*Pv*LDH complex with hLDH-A reveals that a protruding loop and shallow cancave region of hLDH-A is not complementary to convax area of pL1. Yellow stick represents pL1. Marine surface indicates hLDH-A surface. (**B**) Superimposition of the *Pv*LDH:pL1 complex with hLDH-B reveals that the shallow concave region of hLDH-B is not complementry to the convax area of pL1. pL1 is shown as a yellow stick model. hLDH-B is shown as a light pink surface.

Superimposition of the 2008s:*Pf*LDH complex with hLDH reveals shape complementarity between 2008s and hLDH (Fig. S7). However, superimposition of the pL1:*Pv*LDH complex with hLDH shows steric clashes between pL1 and hLDH. Tight packing of the pL1:*Pv*LDH interface is evident because of the hairpin-bulge contact that confers a close approach to the concave *Pv*LDH region on the unpaired nucleotides A11, T12 and T13. Superimposition of pL1-bound *Pv*LDH with hLDH reveals that the concave region of hLDH is shallower than that of *Pv*LDH. Moreover, protruding regions of hLDH aid in the steric clashes between pL1 and *Pv*LDH. The protruding Arg99, Gln100, Gln101 and Y247 of hLDH-A overlap with A11, T12, G23 and A24, respectively. The protruding Arg99, Gln101, Arg112 and Tyr247 of hLDH-B overlap with A11, T12 and G23 of pL1, respectively (Fig. 7). As a result, the shape of pL1 is not complementary to the shallowly concave and protruding region of hLDH, clashing with pL1 binding to hLDH. Therefore, shape complementarity plays a critical role in the specific discrimination of pLDH from hLDH by pL1. In conclusion, the specific protein recognition mode of pL1 appears to be completely different from that of 2008s.

### Structural analysis of deleterious mutations on pL1

Mutations in the set of conserved sequences between pL1 and pL2 dramatically decrease the affinity between pL1 and *Pv*LDH^37^ (Table S7). We designed base substitution mutations to understand the critical roles of the three novel structural elements for *Pv*LDH recognition. In the bulge mutant, the DNA sequence was changed from 5′-GG-3′ to 5′-AA-3′ at the bulge to understand the importance of the BPh and G·G metal ion zippers. Furthermore, the hairpin mutant was designed by changing A22 to G22 at the hairpin loop because the Watson–Crick A22-T12 pair plays a key role in the DNA T-loop-like motif:hairpin loop complex. In addition to mutations, the presence of magnesium ions is critical for proper pL1 folding and *Pv*LDH recognition. Therefore, EMSA was performed under various conditions (Fig. S8, Table S7). As a result, the disturbance in the hairpin-bulge contact, BPh zipper and DNA G·G metal ion zipper has deleterious effects on *Pv*LDH recognition (Fig. 6). As described above, the hairpin-bulge contact, BPh zipper and DNA G·G metal ion zipper play critical roles in the stabilisation of *Pv*LDH-binding unpaired nucleotides at the bulge and hairpin loop. Therefore, pL1 does not recognise *Pv*LDH without the three novel structural elements because the combination of the three novel structural elements confers the stable shape to the unpaired nucleotides.

Interestingly, the G-C rich stem mutant results in a low affinity between pL1 and *Pv*LDH, although the secondary structure of Stem II is maintained (Fig. S8B). The stability of coaxial stacking depends on the sequence of the helix–helix interface. In pL1, coaxial stacking occurs at the helix–helix interface between the Watson–Crick A6-T31 pair and the Watson–Crick G16-C30 pair. In the G-C rich stem mutant, the Watson–Crick A6-T31 pair stacks below the Watson–Crick A16-T30 pair at the helical interface. Base-stacking interactions are stabilised to a greater extent by the Watson–Crick G-C pair than by the Watson–Crick A-T pair^46^,^47^. As a result, pL1 has more stable coaxial stacking than the G-C rich stem mutant. Because coaxial stacking is the main scaffold of pL1 for maintaining other structures, the G-C rich stem mutation reduces the affinity between pL1 and *Pv*LDH. Considering all mutations, it is conceivable that mutations in nucleotides involved in structural elements cause a critical malfunction in the interaction between pL1 and *Pv*LDH.

## Methods

### Purification of recombinant *Pv*LDH and oligo DNA preparation

*Pv*LDH gene sequence was inserted into the pET28a (Novagen) vector and then recombinant *Pv*LDH was transformed into *E. coli.* strain ER2566. Bacteria were incubated at 37 degrees in Luria-Bertani (LB) broth until OD_600_ = 0.5. Then, the recombinant *Pv*LDH was expressed by adding 0.2 mM isopropyl β-d-1-thiogalactopyranoside (IPTG) for 4 hours. Cells were harvested using centrifugation at 5,000 rpm for 10 min. Cells suspended in resuspension buffer (20 mM Tris HCl pH 7.5 and 500 mM NaCl) were sonicated. Then, cell debris was removed by centrifugation at 13,000 rpm for 1 hour. Supernatants were loaded onto a 5-ml Histrap column (GE Healthcare Life Science) and eluted in an elution buffer (20 mM Tris HCl pH 7.5, 500 mM NaCl, and 300 mM imidazole). The elution samples were thereafter passed over Superdex 200 (16/60 GE Healthcare Life Science) in the final buffer containing 20 mM Tris HCl pH 7.5, 50 mM NaCl, 5% (w/v) glycerol, and 5 mM β-mercaptoethanol to remove aggregates. DNA aptamer pL1 with the sequence 5′-GTTCGATTGGATTGTGCCGGAAGTGCTGGCTCGAAC-3′ was synthesised using MOPC^TM^ purification at Macrogen (Seoul, Korea). Oligonucleotides were placed in the binding buffer (20 mM Tris HCl pH 7.5, 50 mM NaCl, 5 mM KCl, and 5 mM MgCl_2_). For proper folding, oligonucleotides were denatured at 90 degrees for 3 minutes and then incubated at 4 degrees for 1 hour.

### Complex formation and crystallization

Recombinant *Pv*LDH and DNA aptamer in the buffer (20 mM Tris HCl pH 7.5, 50 mM NaCl, 2.5 mM KCl, 2.5 mM MgCl_2_, 2.5% (w/v) glycerol, 2.5 mM β-mercaptoethanol) were mixed in a 1:2 ratio at 15 mg/ml of protein concentration. Crystallization was solely performed by sitting-drop vapor-diffusion method at 20 degrees. The needle-shaped crystals of the pL1:*Pv*LDH complex were obtained by mixing 1 μl of the pL1:*Pv*LDH complex with 1 μl of the reservoir solution (0.1 M HEPES pH 7.0 and 15% (w/v) PEG-20 K) after 1 month. To get high-resolution data, additive screening was performed. Then, rod- or octahedron-shaped crystals were grown by mixing 2 μl of the pL1:*Pv*LDH complex with 2 μl of the crystal solution containing 0.1 M HEPES pH 7.0 and 15% (w/v) PEG-20 K with the addition of 0.3 M NDSB-195 or 5% (w/v) PEG-400, respectively, for 1 month. Crystals of apo-*Pv*LDH were observed by mixing 1 μl of the pL1:*Pv*LDH complex with 1 μl of the precipitant containing 0.2 M ammonium nitrate and 20% (w/v) PEG-3350 after 2 months.

### Data collection and processing

Native diffraction data of the pL1:*Pv*LDH complex and apo-*Pv*LDH were collected at the beamline of 5C of Pohang Accelerator Laboratory at a wavelength of 0.9796 Å by using cryo-cooled crystals at 100 K with cryoprotectants involving 30% (w/v) glycerol or 50% (w/v) PEG-20 K. Data processing was achieved with HKL2000 software^48^. The high-resolution cutoff of a dataset was performed according to rules of three for determining resolution limit (1. completeness > 70%, 2. *R*_merge_ (a.k.a *R*_symm_, *R*_linear_) < 0.30 (or 30%), *I*/σ (*I*) > 3). In spite of a conservative cut for *I*/σ (*I*) and completeness, the resolution limits were determined for appropriate *R*_merge_ values. Crystallographic data statistics are described in Table S1.

### Structure determination and refinement

Phases of the pL1:*Pv*LDH complex and apo-*Pv*LDH were determined by phaser-MR in PHENIX package^49^. Monomeric *Pv*LDH (PDB code: 2A92) was used as a molecular replace search model. DNA nucleotides were manually and gradually built into the difference density map with coot^50^. Thereafter, model structures were refined with phenix.refine in PHENIX package^49^. XYZ coordinates, real-space, rigid body, individual ADPs, and occupancies were selected for refinement strategies in common. Non-crystallographic symmetry (torsion-angle restraints type) was used only for the refinement of apo-*Pv*LDH. The TLS refinement strategy was selected only for the pL1:*Pv*LDH complex (PDB code: 5HRU). TLS groups were automatically found by TLSMD server. Secondary structure restraints were essentially used for the refinement of DNA owing to distance restraints for hydrogen bonding interactions in Watson–Crick base pairs. Model building and refinement were iteratively performed to get the best model of the pL1:*Pv*LDH complex and apo-*Pv*LDH. Amino acids approximately from 83 to 95 and nucleotides of 1, 2, 35, and 36 were not observed in the final modes of apo-*Pv*LDH and pL1:*Pv*LDH complexes owing to weak electron density. The *B*-factors for DNA are considerably higher than those for proteins in DNA:protein complexes. In detail, the *B*-factors for solvent-exposed nucleotides are higher than those for protein-interacting nucleotides^51^. Moreover, the RNA backbone is significantly more flexible than that of protein in RNA:protein complexes^52^. A comparison of the *B*-factors in protein:DNA aptamer complexes shows that the *B*-factors for DNA aptamers are significantly higher than those for proteins (Table S8). Therefore, the *B*-factors for nucleic acids are generally higher than those of protein in the complexes. The pL1:*Pv*LDH complex also follows this general trend (Table S1).

### Non-radioactive EMSA

DNA probes (pL1 and pL1 mutants) were synthesised using OPC purification at Bionics (Seoul, Korea). Oligonucleotides were dissolved in the binding buffer or binding buffer without magnesium ions. For proper folding, oligonucleotides were denatured at 90 degrees for 5 minutes and incubated at 4 degrees for 1 hour. Next, 10 pmol of oligonucleotides was mixed with 200 pmol *Pv*LDH or hLDH (Novoprotein) for 1 hour at room temperature in the EMSA buffer (20 μl) or EMSA buffer without MgCl_2_. The EMSA buffer contains 20 mM HEPES pH 8.0, 5% (w/v) glycerol, 50 mM NaCl, 5 mM KCl, 5 mM MgCl_2_, and 5 mM DTT. Samples were loaded into the wells of 10% polyacrylamide gel. The gel was run at 120 V for 1 hour at 4 degrees in 0.5× TBE buffer pH 8.5 containing 45 mM Tris-borate and 1 mM EDTA. DNA and protein molecules were stained with SYBR Green I and Coomassie Brilliant Blue G-250, respectively.

### Figure generation and structural analysis

Figures have been generated using PyMol (http://www.pymol.org), Jmol (http://www.jmol.org) and CCP4MG^53^. Ramachandran plot analysis was performed by RAMPAGE webserver (http://mordred.bioc.cam.ac.uk/~rapper/rampage.php)^54^. Interfaces between DNA aptamers and proteins were analyzed with PyMol, PISA, COCOMAPS web server (https://www.molnac.unisa.it/BioTools/cocomaps/index.p) and CONTACT program from the CCP4 suite^55^. Protein shapes were determined by Concave Finder employing modified Cx algorithm (http://cib.cf.ocha.ac.jp/bitool/CONCAVE)^42^. The magnesium ion position was validated by CheckMyMetal web server (http://csgid.org/csgid/metal_sites)^56^. All parameters were acceptable for magnesium ions (Fig. S3, Table S2). The secondary structures of pL1 mutants were confirmed using mfold web server (http://www.bioinfo.rpi.edu/applications/mfold)^57^. Sc values were calculated with Sc software^58^ from the CCP4 suite of programs^54^. Sc_radii.lib file was modified owing to the absence of the van-der-Waals radii for nucleic acid atoms. Atomic radii for proteins were set according to the default in the sc_radii.lib file. Atomic radii for DNA were set according to those for chemically similar atoms of proteins.

### Data availability

Apo-PvLDH and two forms of pL1:PvLDH complexes were deposited in the Protein Data Bank under PDB ID codes: 5HS4, 5HRU and 5HTO, respectively.

## Additional Information

**How to cite this article**: Choi, S.-J. and Ban, C. Crystal structure of a DNA aptamer bound to *Pv*LDH elucidates novel single-stranded DNA structural elements for folding and recognition. *Sci. Rep.* **6**, 34998; doi: 10.1038/srep34998 (2016).

## Supplementary Material

Supplementary Information

## Figures and Tables

**Table 1:** Analysis of interfaces between proteins and DNA aptamers.

Protein: aptamer (PDB code)	*Pv*LDH: pL1 (5HRU)	*Pf*LDH: 2008s (3ZH2)	thrombin: mTBA (3QLP)	thrombin: TBA (4DIH)	thrombin: RE31 (5CMX)	ATX: RB011 (5HRT)	VWF: ARC1172 (3HXO)	HIV-1 RT: atp-DNA (5I3U)
ΔASA	1145.2	1317.1	570	563.25	552.7	934.6	1014.25	2054.15
H-bonds	5	15	11	9	9	12	24	19
H-bonds (/100 Å^2^ ΔASA)	0.4	1.13	1.9	1.59	1.62	1.28	2.07	0.92
Bridging waters	16	3	5	4	2	5	0	0
Bridging waters (/1000 Å^2^ ΔASA)	13.9	2.2	7	7.1	3.6	5.3	0	0

The data for thrombin:DNA aptamer complexes are obtained from a previous interface analysis^31^,^34^.
