# Supplementary material for: Crystal structure of a DNA aptamer bound to PvLDH elucidates novel single-stranded DNA structural elements for folding and recognition
Source: Sci Rep. 2016 Oct 11;6:34998. doi: 10.1038/srep34998 (PMC5057103; doi:10.1038/srep34998)
Supplement: Supplementary Information [file srep34998-s1.pdf]

Supplementary Information

Crystal structure of a DNA aptamer bound to *Pv*LDH elucidates novel single-stranded DNA structural elements for folding and recognition

Sung-Jin Choi & Changill Ban*

Department of Chemistry, Pohang University of Science and Technology (POSTECH), Pohang, 790-784, South Korea

*Correspondence and requests for materials should be addressed to C. Ban (email: ciban@postech.ac.kr)

SUPPLEMENTARY FIGURES

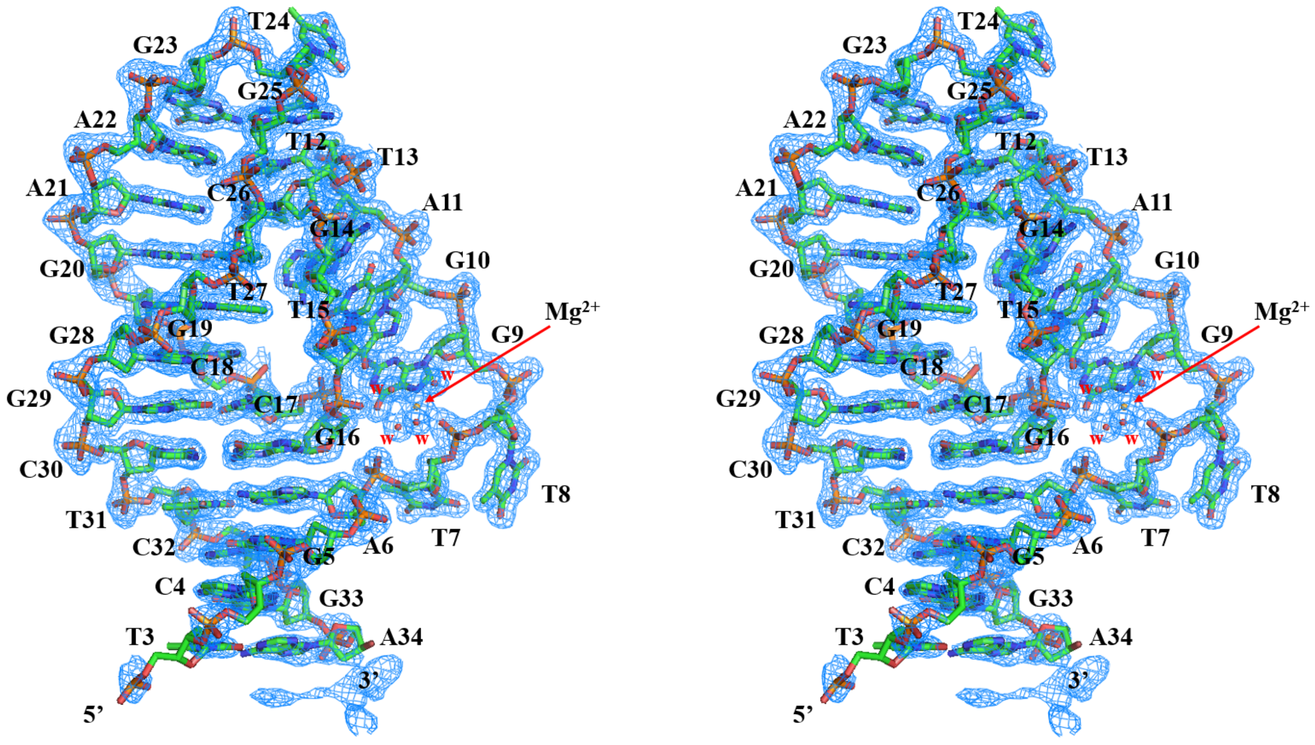

Supplementary Figure S1. The *2mFo-DFc* electron density map of a hydrated magnesium ion-bound pL1 contoured at 1.0 σ is shown as a cyan mesh (Wall-eyed stereo images). pL1, magnesium ion and inner-sphere waters are shown as cyan stick, gold sphere and red spheres, respectively.

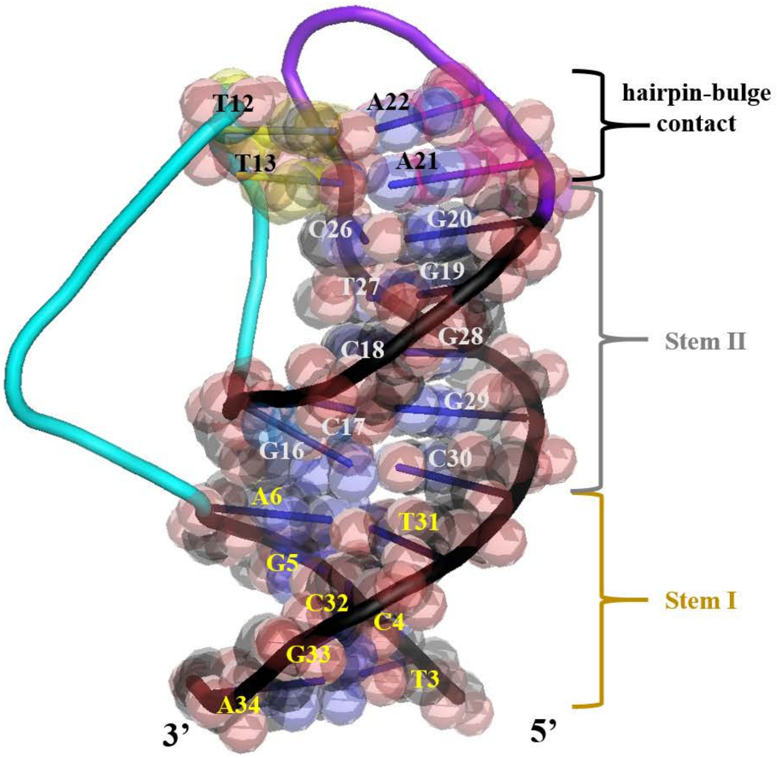

Supplementary Figure S2. One pseudo-continuous helix is formed from three stems by base stacking. Strands forming the bulge, hairpin loop, and stems are shown in cyan, purple, and black, respectively. Single letter codes of nucleotides at Stem I, Stem II, and the hairpin-bulge contact are colored in yellow, grey, and black, respectively.

A

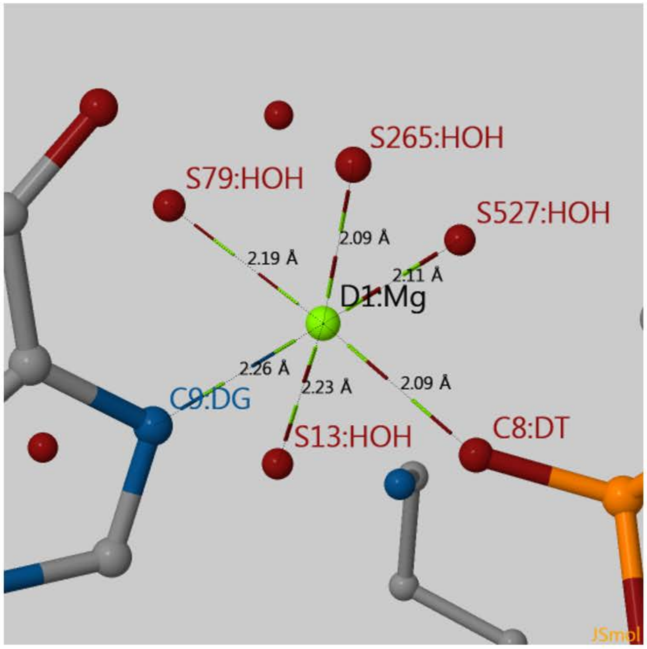

B

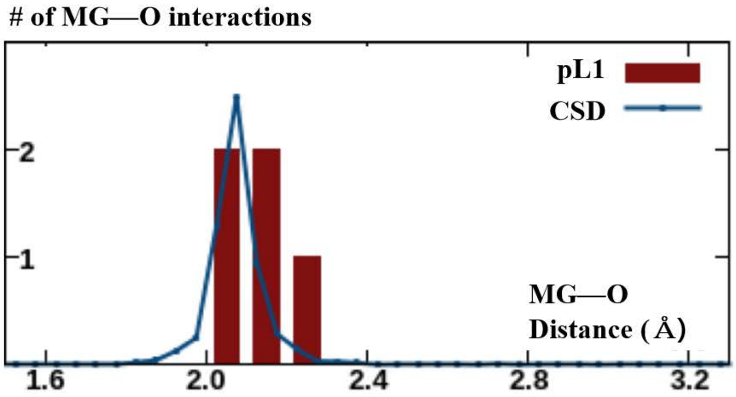

CSD: Cambridge Structural Database

Supplementary Figure S3. Mg—O distance and validation of the magnesium ion-binding site for pL1 (PDB code: 5HRU) with CheckMyMetal web server. See also Table S2. (A) Mg—O distance in the inner-sphere of the magnesium ion for pL1 (PDB code: 5HRU) (B) Mg—O distance distribution for pL1 (PDB code: 5HRU) in comparison with CSD.

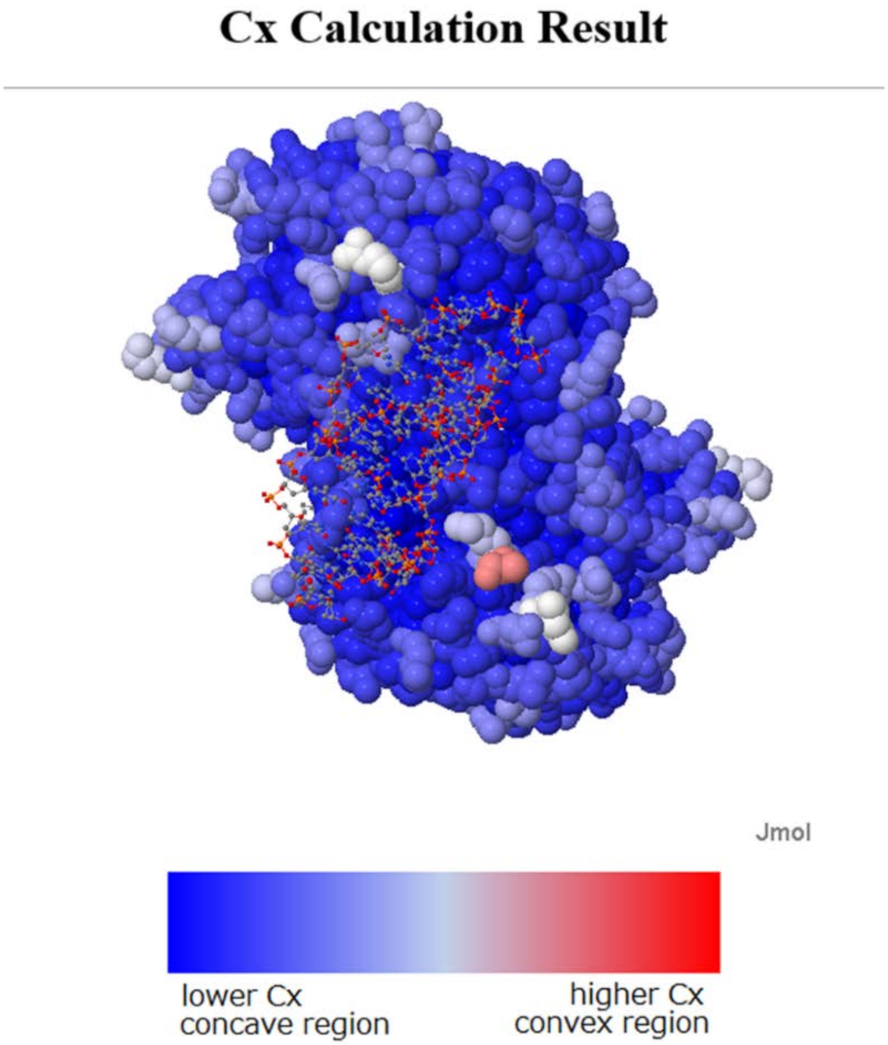

Supplementary Figure S4. Cx calculation result of *Pv*LDH in complex with pL1. DNA aptamer pL1 is shown as ball and stick. *Pv*LDH is shown as spheres, with colors corresponding to the Cx values[1]. Blue and red indicate the concave region and convex region, respectively.

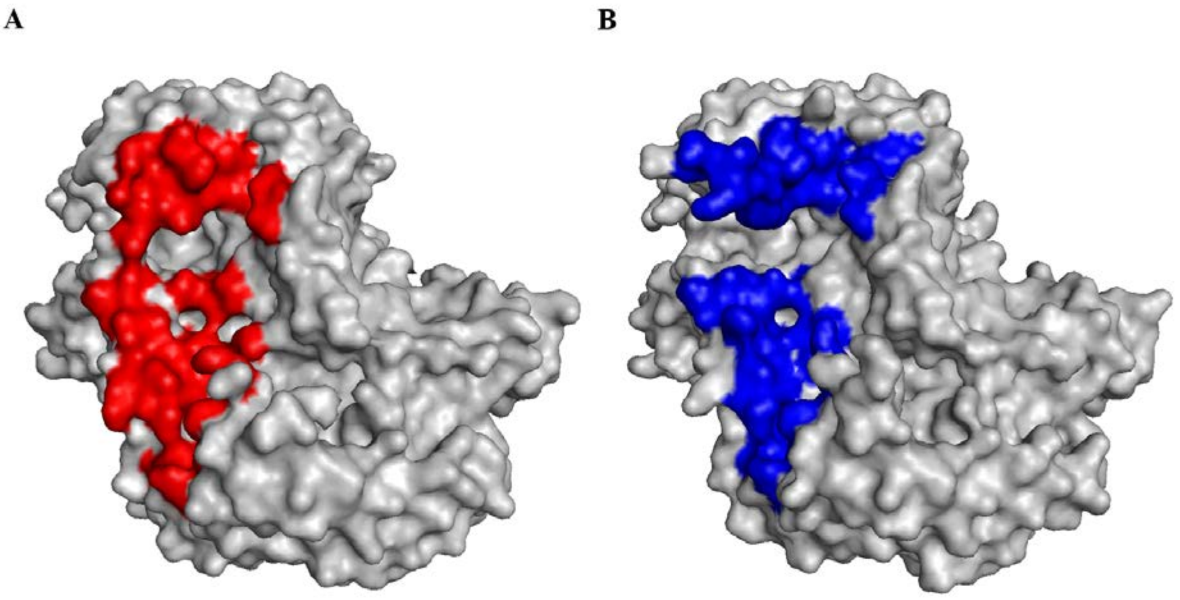

Supplementary Figure S5. DNA aptamer-contacting residues of *Plasmodium* LDH in the same orientation. (**A**) Red pL1-contacting residues of *Pv*LDH show a single binding head. (**B**) Blue 2008s-contacting residues of *Pf*LDH display a double binding head.

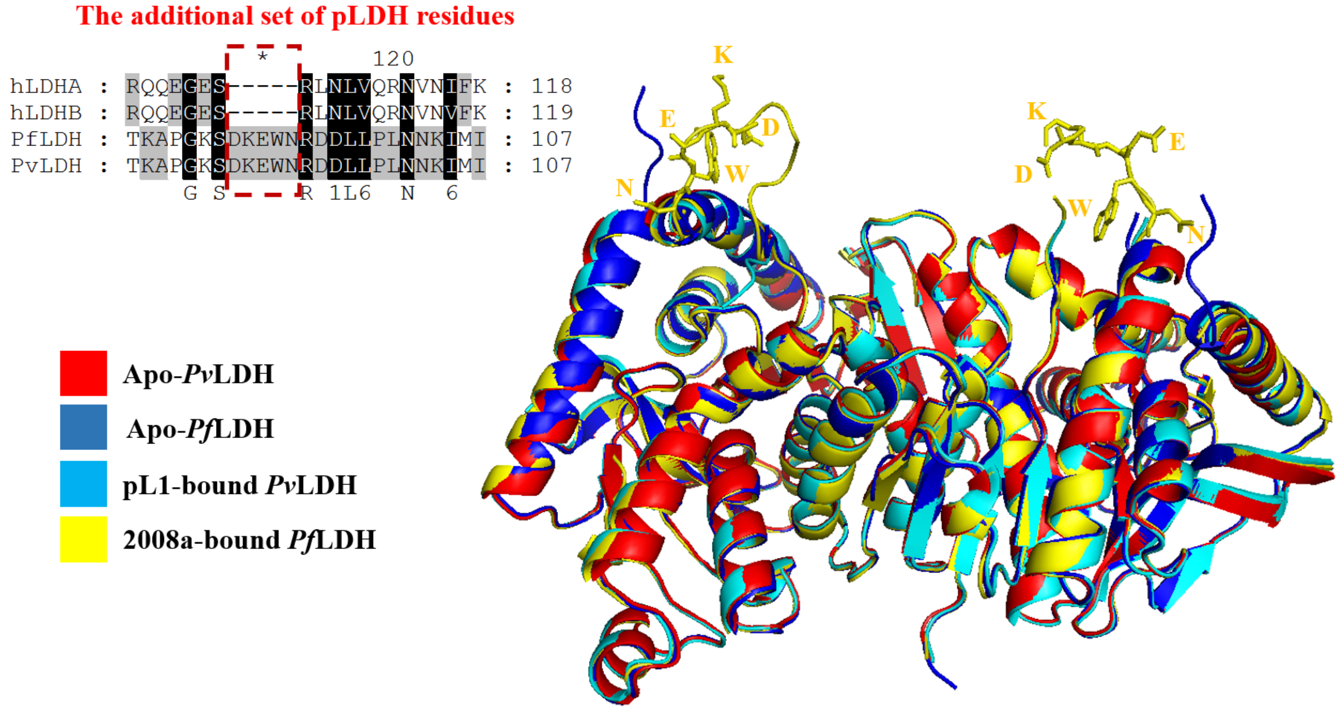

Supplementary Figure S6. Superimposition of apo-*Pv*LDH (PDB code: 5HS4) with apo-*Pf*LDH (PDB code: 2X8L), pL1-bound *Pv*LDH (PDB code: 5HRU), and 2008s-bound *Pf*LDH (3ZH2). All proteins are represented as cartoon. Additional residues of pLDH are shown as stick. Apo-*Pv*LDH, apo-*Pf*LDH, pL1-bound *Pv*LDH, and 2008s-bound *Pf*LDH are shown in red, blue, cyan, and yellow, respectively. The loop (FL) containing additional pLDH amino acids is disordered in apo-*Pv*LDH, apo-*Pf*LDH and pL1-bound *Pv*LDH.

(A) (B)

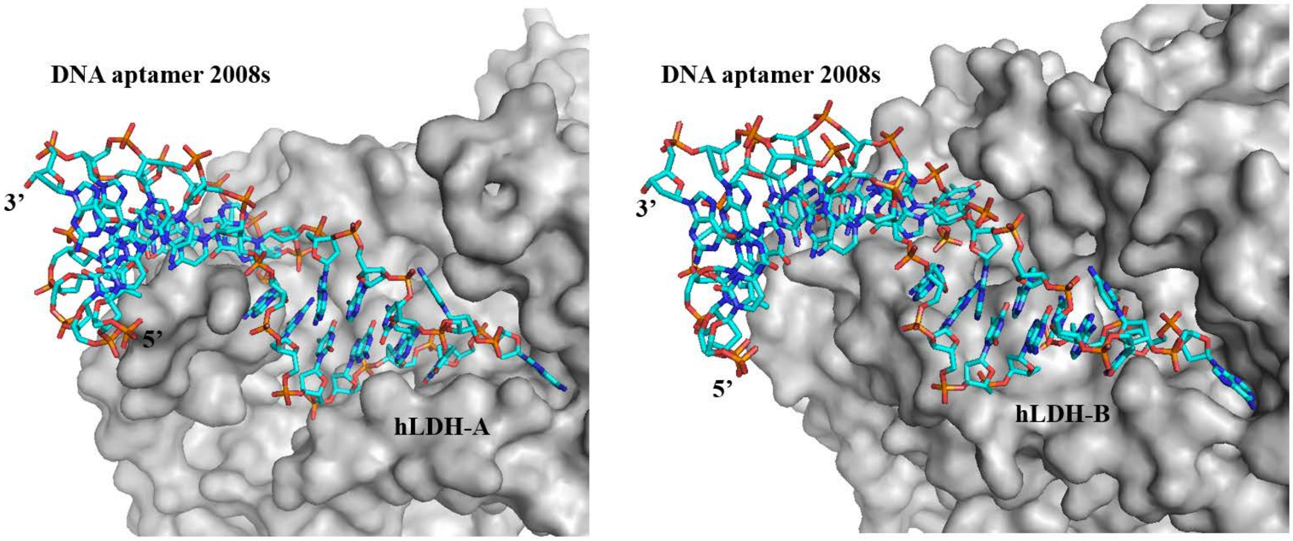

Supplementary Figure S7. Shape complementarity between 2008s and hLDH. No steric clashes are observed between 2008s and hLDH. (A) Superimposition of the 2008s:*Pf*LDH complex with hLDH-A (PDB code: 4OJN). (B) Superimposition of the 2008s:*Pf*LDH complex with hLDH-B (PDB code:1T2F).

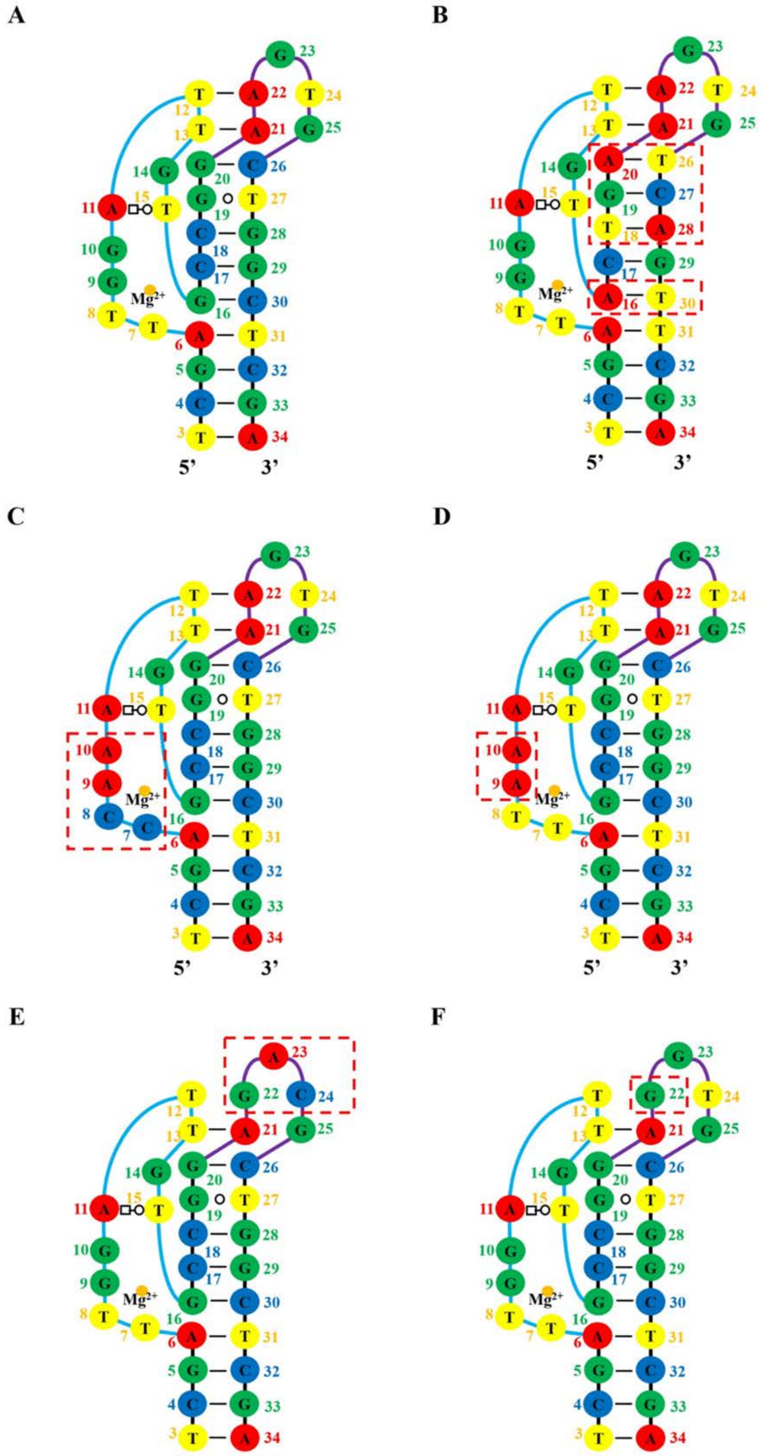

Supplementary Figure S8. Secondary structures of pL1 mutants derived from the tertiary structure of pL1 and mfold web server[2]. Altered nucleotides from pL1 are boxed with a dashed red line. (**A**) Secondary structure of pL1 derived from the tertiary structure of pL1. (**B**) Secondary structure of the G-C rich stem mutant. (**C**) Secondary structure of the loop 1 mutant. (**D**) Secondary structure of the bulge mutant. (**E**) Secondary structure of the loop 2 mutant. (**F**) Secondary structure of the hairpin loop mutant.

SUPPLEMENTARY TABLES

Supplementary Table S1. Crystallographic data collection and refinement statistics. Statistics for the highest-resolution shell are shown in parentheses. Ramachandran plot statistics were calculated by RAMPAGE web server.

	Apo-*Pv*LDH	pL1:*Pv*LDH (dimeric)	pL1:*Pv*LDH (tetrameric)
**Data collection**			
Space group	*I*222	$P4_12_12$	$P2_12_12_1$
Cell dimensions			
a, b, c (Å)	75.7, 80.6, 93.3	84.5, 84.5, 209.9	85.5, 86.5, 209.1
α, β, γ (°)	90.0, 90.0, 90.0	90.0, 90.0, 90.0	90.0, 90.0, 90.0
Resolution (Å)	23.77-1.33 (1.38-1.33)	24.96-1.71 (1.73-1.71)	25.00-1.90 (1.96-1.90)
R_{merge}	0.07 (0.26)	0.10 (0.28)	0.08 (0.26)
Mean I / σ (I)	43.64 (11.18)	49.31 (11.41)	45.27 (14.84)
Completeness (%)	99.0 (95.8)	99.8 (99.3)	99.9 (99.23)
Redundancy	11.8 (9.0)	21.4 (15.4)	14.6 (14.4)
**Refinement**			
No. unique reflections	63,845 (6,093)	82,462 (8,064)	122,577 (12,020)
R_{work} / R_{free}	0.14 / 0.17	0.15 / 0.18	0.17 / 0.20
No. atoms			
Protein	2292	4634	9202
DNA / ion	N/A	663 / 1	1203 / 2
Water	223	526	903
Wilson *B*-factor	13.6	20.3	17.9
B-factors			
Overall	17.8	24.5	20.1
Protein	17.0	21.7	17.3
DNA / ion	N/A	37.2 / 19.5	31.9 / 19.6
Water	26.1	29.7	23.7
r.m.s. deviations			
Bond lengths (Å)	0.008	0.008	0.009
Bond angles (°)	1.16	1.12	1.12
**Ramachandran plot**			
Favored (%)	97.7	97.8	97.8
Allowed (%)	2.0	1.8	1.8
Outlier (%)	0.3	0.3	0.3

Supplementary Table S2. Validation of the magnesium-binding site in pL1 via the CheckMyMetal web server. See also Fig. S3. All descriptions of CheckMyMetal parameters are taken from CheckMyMetal web server. All parameters are acceptable for the magnesium ion-biding site without alternative metal ions and bidentate.

ID	Res.	Metal	*Occup-ancy*	*B factor (env.)*[3]	*Ligands*	*Valence*[4]	*nVECSUM*[5]	Geometry[3,6]	*gRMSD* (°)[3]	Vacan-cy[3]	Bident-ate	Alt. metal
D:1	MG	Mg	1	19.6 (20.9)	O_5N_1	1.7	0.052	Octahedral	2.5°	0	0	

Legend:	Not applicable	**Outlier**	*Borderline*	Acceptable

Column	Description
Occupancy	Occupancy of ion under consideration.
B factor (env.)[3]	Metal ion B factor, with valence-weighted environmental average B factor in parenthesis.
Ligands	Elemental composition of the coordination sphere.
Valence[4]	Summation of bond valence values for an ion binding site. *Valence* accounts for metal-ligand distances.
nVECSUM[5]	Summation of ligand vectors, weighted by bond valence values and normalized by overall valence. Increase when the coordination sphere is not symmetrical due to incompleteness.
Geometry[3,6]	Arrangement of ligands around the ion, as defined by the *NEIGHBORHOOD* algorithm.
gRMSD(°)[3]	R.M.S. Deviation of observed geometry angles (L-M-L angles) compared to ideal geometry, in degrees.
Vacancy[3]	Percentage of unoccupied sites in the coordination sphere for the given geometry.
Bidentate	Number of residues that form a bidentate interaction instead of being considered as multiple ligands.
Alt. metal	A list of alternative metal(s) is proposed in descending order of confidency, assuming metal environment is accurately determined. This feature is still experimental. It requires user discrimination and cannot be blindly accepted.

Supplementary Table S3. Interactions between pLDH and DNA aptamers within 4 Å. Red resides are involved in salt bridge interactions. Bold protein residues are implicated in the recognition of both 2008s and pL1.

	*Pv*LDH:pL1 (5HRU)		*Pf*LDH:2008s (3ZH2)	
	Protein residues	Aptamer residues	Protein residues	Aptamer residues
Hydrogen bonding interaction			Asp35 (OD1) chain A	T9 (N3)
			Ile36 (N) chain A	T9 (O4)
	**Lys44 (NZ) chain B**	G10 (OP1)	**Lys44 (NZ) chain B**	A18 (OP1)
	Gly81 (N) chain B	T8 (O3’)		
	Thr83 (O) chain A	G23 (N1, N2)		
				A10 (OP2), A22 (N1)
			Lys84 (NZ) chain A	A22 (N6)
			Ala85 (O) chain A	A22 (OP2)
			Lys88 (NZ) chain A	G11 (N7)
			Lys88 (N) chain A	G11 (O6)
			Ser89 (N) chain A	G11 (O6), C6 (N4)
			Ser89 (OG) chain A	G7 (N1)
			Asp90 (OD1) chain A	G7 (N2), G8 (N2)
			Asp90 (OD2) chain A	T17 (O2)
	Tyr236 (OH) chain A	A11 (OP1)	His232 (ND1) chain A	
Interacting residues	Gly11 chain B	G9		
	**Gly13 chain B**	G9, G10	**Gly13 chain B**	A18, T17
	**Met14 chain** A; **B**	A11; G10	**Met14 chain B**	A18
			Phe34 chain A	T9
	**Asp35 chain** B	G9	**Asp35 chain** A	T9
	**Val36 chain A; B**	T24; T7, T8	**Ile36 chain A; B**	T9; A16
	**Val37 chain** A; **B**	T24; G9	**Val37 chain B**	T17
	Lys38 chain B	T7		
	**Met40 chain B**	G9	**Met40 chain B**	T17
	**Lys44 chain B**	G10	**Lys44 chain B**	T17, A18,
			Tyr67 chain A	T9
	Thr79 chain B	G9		
	**Ala80 chain B**	G9, T8	**Ala80 chain** A; **B**	T9; A16
	**Gly81 chain A; B**	T24; T8	**Gly81 chain A; B**	T9; A16
	**Phe82 chain A; B**	G25, T24, G23; T8	**Phe82 chain A; B**	T9, A10; A16
	Thr83 chain A	T8		
	**Lys84 chain A**	G23, T12, G23	**Lys84 chain A**	A10, A22, G21
			Ala85 chain A	A22
			Pro86 chain A	A10, G8, A22
			Gly87 chain A	G11, G7, A10, A22
			Lys88 chain A	C23, A22, G11
			Ser89 chain A	C6, G11, G7, G5, C23
			Asp90 chain A	G8, C6, G7
			Lys91 chain A	G5, C23, G4
	Asp97 chain A	G23		
	Leu98 chain A	G23		
	Leu101 chain A	G23		
	**Ile105 chain A; B**	T24; T8	**Ile105 chain A; B**	T9; A16
			Glu108 chain B	A16
			Val229 chain A	T20, G19
	**Leu232 chain A**	C18, C17, G9	**His232 chain A**	T17, G19, A18
			Ala233 chain A	G19
	**Ser234 chain A**	A11	**Ser234 chain A**	G19
	Tyr236 chain A	A11		

Supplementary Table S4. Interface properties between proteins and nucleic acids. The data for protein:RNA, protein:DNA, and protein:protein are taken from a previous interface analysis[7,8]. Protein:DNA aptamer complexes include thrombin:mTBA (PDB code: 3QLP), thrombin:TBA (PDB code: 4DIH), thrombin:RE31 (PDB code: 5CMX), HIV-1 RT:apt-DNA (PDB code: 5I3U), ATX:RB011 (5HRT), *Pf*LDH:2008a (3ZH2), and VWF:ARC1172 (3HXO) complexes. The parameters are described below. ΔASA: Half of the buried ASA (Å^2) within 5 Å upon complex formation was calculated using COCOMAPS web server employing NACCESS. Hydrogen bonds (/100 Å^2 ΔASA): Number of intermolecular hydrogen bonds per Å^2 ΔASA was calculated using COCOMAPS web server employing NACCESS. Bridging waters (/100 Å^2 ΔASA): Number of water molecules forming hydrogen bonds with both DNA aptamers and proteins per 1000 Å^2 ΔASA was calculated using CANTACT.

	Protein: RNA	Protein: DNA	Protein: Protein	Protein: DNA aptamer	*Pv*LDH: pL1
No. of complexes	89	115	115	7	1
ΔASA	1230	1568	943	1008.4	1145
Bridging waters (/1000 Å^2 ΔASA)	8.1	9.6	5.4	2.49	13.9
No. of complexes	20	29	59	7	1
Hydrogen bonds (/100 Å^2 ΔASA)	1.2	1.3	0.8	1.40	0.4

Supplementary Table S5. Bridging waters between *Pv*LDH and pL1 (PDB code: 5HRU).

pL1<br>Atom (residue)	Distance (Å)<br>(pL1 to Water)	Water molecule<br>Number	Distance (Å)<br>(Water to *Pv*LDH)	*Pv*LDH<br>Atom (residue)
OP2 (C17)	2.69	5	2.86	O (Lys38 B)
N7 (G9)<br>O2 (T8)<br>OP1 (T8)	3.15<br>2.67<br>3.11	13	2.67	NZ (Lys84 B)
O2 (T8)	2.67	19	2.81	OH (Tyr67 B)
N2 (G9)<br>O4′ (G10)	2.84<br>2.98	34	2.65	O (Leu232 A)
N3 (T8)	2.86	37	2.77<br>2.86	OH (Tyr67 B)<br>OE1 (Glu108 B)
OP2 (G9)	3.07	49	2.96<br>3.28	N (Val36 B)<br>OD1 (Asp35 B)
OP1 (G9)	2.61	84	2.89<br>2.70	N (Gly13 B)<br>OG1 (Thr79 B)
OP2 (T8)	2.74	99	2.61	NZ (Lys84 B)
N3 (G10)	2.91	139	3.28	O (Leu232 A)
O4′ (T12)<br>N2 (G23)<br>O6 (G25)	3.21<br>2.93<br>2.76	161	2.82<br>2.86	N (Thr83 A)<br>OG1 (Thr83 A)
OP1 (A11)	2.70	188	3.13	N (Ser234 A)
OP1 (T12)	2.70	194	3.02	OG (Ser234 A)
O2 (T24)<br>N3 (C24)	3.02<br>2.66	248	2.71	OD1 (Asp35 A)
OP1 (T12)	2.44	282	3.09	N (Met14 A)
O6 (G25)	2.63	344	3.09	O (Gly81 A)
OP1 (C18)<br>OP2 (C18)	3.09<br>3.17	354	2.83	O (Asn230 A)

Supplementary Table S6. Shape complementarity analysis among pLDH:DNA aptamer complexes with Sc software from the CCP4 suite of programs[9]. Sc values are the quantitative shape complementarity indices between two surfaces. A maximum value of 1.0 shows perfect shape complementarity. The Sc values are independent on buried surface areas[10]. pL1:*Pv*LDH complex (PDB code: 5HRU) and 2008:*Pf*LDH complex (PDB code: 3ZH2) were used for Sc calculation. pLDHW indicates pLDH with bridging waters. pLDH shows pLDH without bridging waters. pLDHWwoFL presents that FL is not considered in pLDHW.

Aptamer: pLDH	2008a: *Pf*LDH	pL1: *Pv*LDH	2008a: *Pf*LDHW	pL1: *Pv*LDHW	2008a: *Pf*LDHWwoFL	pL1: *Pv*LDHWwoFL
Sc	0.743	0.675	0.744	0.720	0.685	0.717

Supplementary Table S7. Sequences of pL1 and its mutants. The altered nucleotides from pL1 and underlined. Sequences of the G-G rich tem, loop 1, and loop 2 mutants are taken from previous mutation studies[11].

ssDNA	Sequence (5′ to 3′)
pL1	GTTCGATTGGATTGTGCCGGAAGTGCTGGCTCGAAC
G-C rich stem mutant	GTTCGATTGGATTGTACTGAAAGTGTCAGTTCGAAC
Loop 1 mutant	GTTCGACCAAATTGTGCCGGAAGTGCTGGCTCGAAC
Bulge mutant	GTTCGATTAAATTGTGCCGGAAGTGCTGGCTCGAAC
Loop 2 mutant	GTTCGATTGGATTGTGCCGGAGACGCTGGCTCGAAC
Hairpin mutant	GTTCGATTGGATTGTGCCGGAGGTGCTGGCTCGAAC

Supplementary Table S8. *B*-factors for proteins and DNA aptamers in protein:DNA aptamer complexes. Average *B*-factors are taken from a previous analysis[12-16] or calculated with PyMol.

Protein: aptamer (PDB code)	*Pv*LDH: pL1 (5HRU)	*Pf*LDH: 2008a (3ZH2)	thrombin: mTBA (3QLP)	thrombin: TBA (4DIH)	thrombin: RE31 (5CMX)	ATX: RB011 (5HRT)	VWF: ARC1172 (3HXO)	HIV-1 RT: atp-DNA (5I3U)
B-factor (protein)	21.7	25.7	18.29	25.6	55.74	38.3	49.8	102.0
B-factor (DNA)	37.2	36.5	25.9	38.0	55.5	53.2	95.9	117.0

References

1 Pintar, A., Carugo, O. & Pongor, S. CX, an algorithm that identifies protruding atoms in proteins. *Bioinformatics* **18**, 980-984 (2002).

2 Zuker, M. Mfold web server for nucleic acid folding and hybridization prediction. *Nucleic Acids Research* **31**, 3406-3415, doi:10.1093/nar/gkg595 (2003).

3 Zheng, H. *et al.* Validation of metal-binding sites in macromolecular structures with the CheckMyMetal web server. *Nature protocols* **9**, 156-170, doi:10.1038/nprot.2013.172 (2014).

4 Brown, I. D. Recent developments in the methods and applications of the bond valence model. *Chemical reviews* **109**, 6858-6919, doi:10.1021/cr900053k (2009).

5 Muller, P., Kopke, S. & Sheldrick, G. M. Is the bond-valence method able to identify metal atoms in protein structures? *Acta crystallographica. Section D, Biological crystallography* **59**, 32-37 (2003).

6 Kuppuraj, G., Dudev, M. & Lim, C. Factors governing metal-ligand distances and coordination geometries of metal complexes. *The journal of physical chemistry. B* **113**, 2952-2960, doi:10.1021/jp807972e (2009).

7 Jones, S., Daley, D. T., Luscombe, N. M., Berman, H. M. & Thornton, J. M. Protein-RNA interactions: a structural analysis. *Nucleic Acids Res* **29**, 943-954 (2001).

8 Barik, A. & Bahadur, R. P. Hydration of protein-RNA recognition sites. *Nucleic Acids Res* **42**, 10148-10160, doi:10.1093/nar/gku679 (2014).

9 Winn, M. D. *et al.* Overview of the CCP4 suite and current developments. *Acta crystallographica. Section D, Biological crystallography* **67**, 235-242, doi:10.1107/S0907444910045749 (2011).

10 Lawrence, M. C. & Colman, P. M. Shape complementarity at protein/protein interfaces. *J Mol Biol* **234**, 946-950, doi:10.1006/jmbi.1993.1648 (1993).

11 Lee, S. *et al.* A highly sensitive aptasensor towards Plasmodium lactate dehydrogenase for the diagnosis of malaria. *Biosens Bioelectron* **35**, 291-296, doi:10.1016/j.bios.2012.03.003 (2012).

12 Cheung, Y. W. *et al.* Structural basis for discriminatory recognition of Plasmodium lactate dehydrogenase by a DNA aptamer. *Proc Natl Acad Sci U S A* **110**, 15967-15972, doi:10.1073/pnas.1309538110 (2013).

13 Russo Krauss, I., Spiridonova, V., Pica, A., Napolitano, V. & Sica, F. Different duplex/quadruplex junctions determine the properties of anti-thrombin aptamers with mixed folding. *Nucleic Acids Res* **44**, 983-991, doi:10.1093/nar/gkv1384 (2016).

14 Kato, K. *et al.* Structural basis for specific inhibition of Autotaxin by a DNA aptamer. *Nature structural & molecular biology* **23**, 395-401, doi:10.1038/nsmb.3200 (2016).

15 Russo Krauss, I. *et al.* High-resolution structures of two complexes between thrombin and thrombin-binding aptamer shed light on the role of cations in the aptamer inhibitory activity. *Nucleic Acids Res* **40**, 8119-8128, doi:10.1093/nar/gks512 (2012).

16 Russo Krauss, I. *et al.* Thrombin-aptamer recognition: a revealed ambiguity. *Nucleic Acids Res* **39**, 7858-7867, doi:10.1093/nar/gkr522 (2011).
